# From descriptive to predictive distribution models: a working example with Iberian amphibians and reptiles

**DOI:** 10.1186/1742-9994-3-8

**Published:** 2006-05-04

**Authors:** JW Arntzen

**Affiliations:** 1 National Museum of Natural History – Naturalis, P. O. Box 9517, 2300 RA Leiden, The Netherlands and Centro de Investigação em Biodiversidade e Recursos Genéticos, Campus Agrário de Vairão, Rua Padre Armando Quintas, 4485-661 Vairão, Portugal

## Abstract

**Background:**

Aim of the study was to identify the conditions under which spatial-environmental models can be used for the improved understanding of species distributions, under the explicit criterion of model predictive performance. I constructed distribution models for 17 amphibian and 21 reptile species in Portugal from atlas data and 13 selected ecological variables with stepwise logistic regression and a geographic information system. Models constructed for Portugal were extrapolated over Spain and tested against range maps and atlas data.

**Results:**

Descriptive model precision ranged from 'fair' to 'very good' for 12 species showing a range border inside Portugal ('edge species', kappa (k) 0.35–0.89, average 0.57) and was at best 'moderate' for 26 species with a countrywide Portuguese distribution ('non-edge species', k = 0.03–0.54, average 0.29). The accuracy of the prediction for Spain was significantly related to the precision of the descriptive model for the group of edge species and not for the countrywide species. In the latter group data were consistently better captured with the single variable search-effort than by the panel of environmental data.

**Conclusion:**

Atlas data in presence-absence format are often inadequate to model the distribution of species if the considered area does not include part of the range border. Conversely, distribution models for edge-species, especially those displaying high precision, may help in the correct identification of parameters underlying the species range and assist with the informed choice of conservation measures.

## Background

Atlases of distribution data have provided a popular and successful way of assembling spatial information on a variety of organisms, including amphibians and reptiles. Most mapping projects are terminated on the production of the atlas while others are continued to improve coverage and to increase spatial and temporal resolution [1-3]. Attempts to analyse and interprete atlases have been infrequent [4,5]. Moreover, the building of distribution models is not always clearly distinguished from the evaluation of the results. These are serious shortcomings because data plots require interpretation before they can be used for i.e., increased biological understanding, policy making and conservation management [6,7]. Even for the best of empirical data sets, the analysis is not straightforward because one has to choose from a wide variety of spatial-statistical analytical techniques, each of which carries assumptions that are unlikely to be met in full [8-10]. Inadequate and uneven sampling, grid cell data notation instead of exact localities, identification error, colinearity, spatial autocorrelation and other non-independencies of the data and statistical artefacts will bias parameter estimates and therewith affect model selection and results [11-15]. More recently, the emphasis has been how species-specific traits such as habitat usage, detectability, prevalence and tolerance may affect model performance [16-18]. In the present paper I aim to analyse distribution patterns of selected species through the joint analysis of distributional and environmental data, first, to construct descriptive distribution models in a GIS (Geographical Information System) environment; second, to evaluate the usefulness of the models for biological interpretation by testing their performance outside the area in which they were generated; and third, to test the hypothesis that low vs. high precision of descriptive models would be associated with low vs. high accuracy in predictive settings. I choose to work with the amphibians and reptiles of the Iberian Peninsula. Whereas the Portuguese herpetofauna is well surveyed in terms of density and spread, mostly by a single, qualified researcher (Mr. R. Malkmus), records for Spain are less dense, unevenly distributed and obtained from a wide variety sources. Hence, the data for Portugal and Spain serve for model constructing and model testing, respectively.

This paper presents distribution models for amphibians and reptiles in Portugal build from environmental variables. The species are classified according to whether their Portuguese distribution includes part of the range border or not. The descriptive performance of both groups of models is compared, finding that the edge species perform significantly better. Alternative models using only search effort as predictor variable perform better than environmental models for countrywide species, whereas the opposite is true for edge species. The predictive performance of edge species' models in Spain was also better than that of countrywide species.

## Results

**Figure 1:**
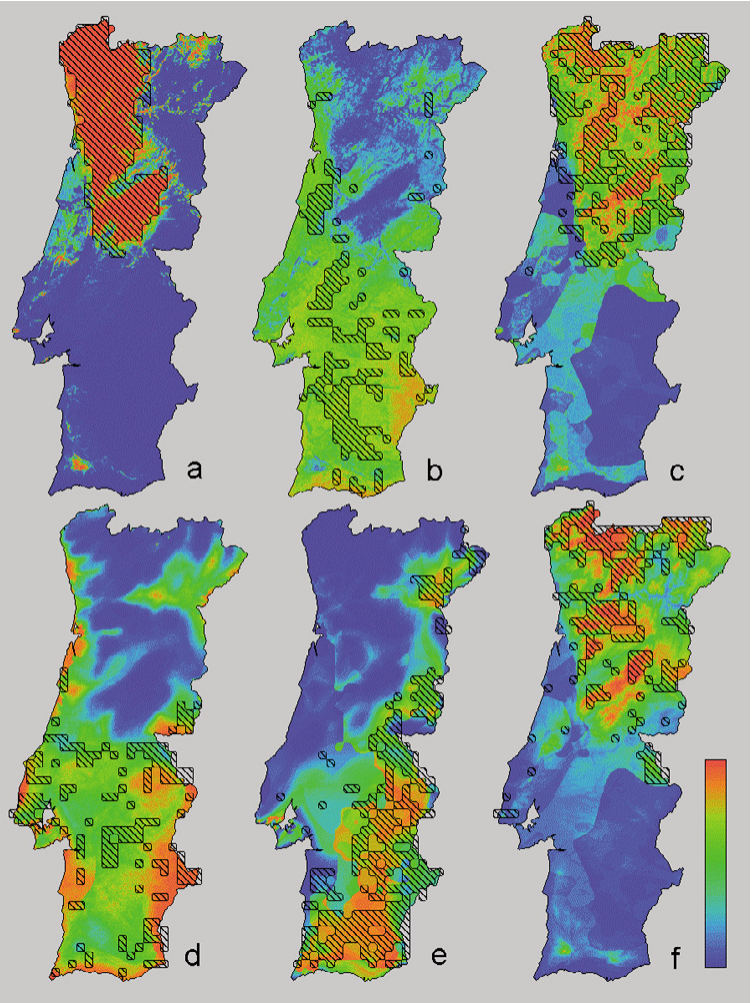
**Descriptive distribution models for six amphibian species across Portugal**. Descriptive distribution models for six amphibian species across Portugal. Models are derived with stepwise logistic regression analysis of the dependent variable 'presence-absence of the target species' against 13 independent ecological variables (details see text and Table 1). The estimated probability of occurrence (g) ranges from 0 (blue) to 1 (red). Composite colours represent intermediate probabilities as in the colour scale bar. Species are: a) *Chioglossa lusitanica*, b) *Pleurodeles waltl*, c) *Triturus marmoratus*, d) *T. pygmaeus*, e) *Alytes cisternasii* and f) *A. obstetricans*. Recorded presences over the 10 × 10 km UTM-grid are shown by black shadings, after Godinho et al. [34].

**Figure 2:**
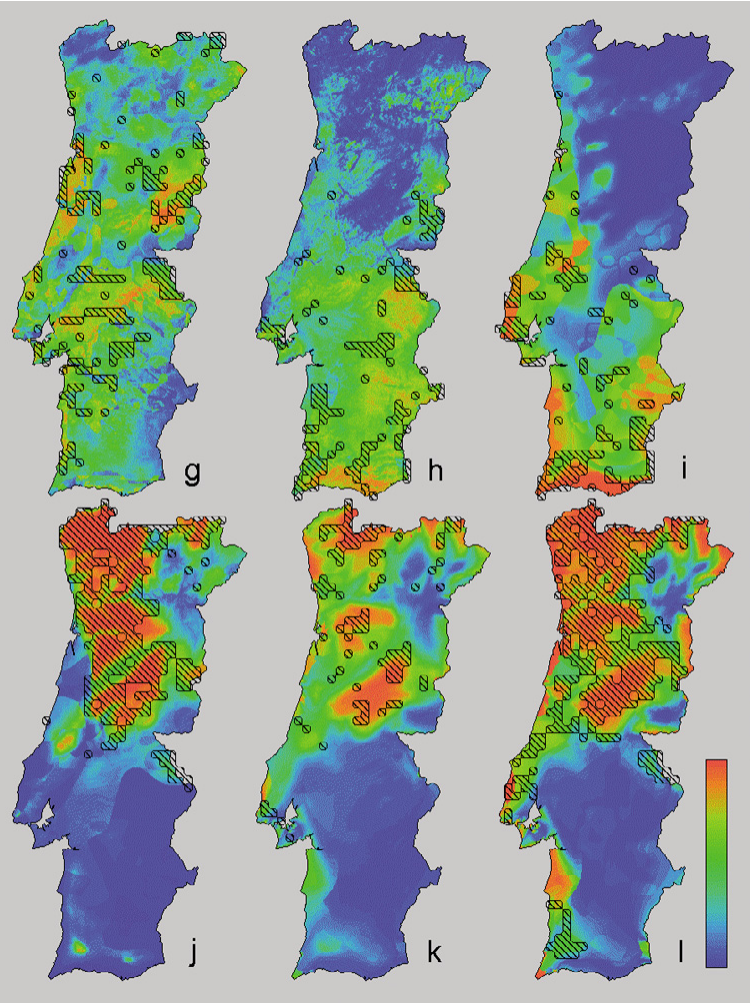
**Descriptive distribution models for four amphibian and two reptile species across Portugal**. As Fig. 1, with four amphibian and two reptile species as follows: g) *Hyla arborea*, h) *H. meridionalis*, i) *Pelodytes ibericus*, j) *Rana iberica*, k) *Anguis fragilis* and l) *Lacerta schreiberi*.

**Figure 3:**
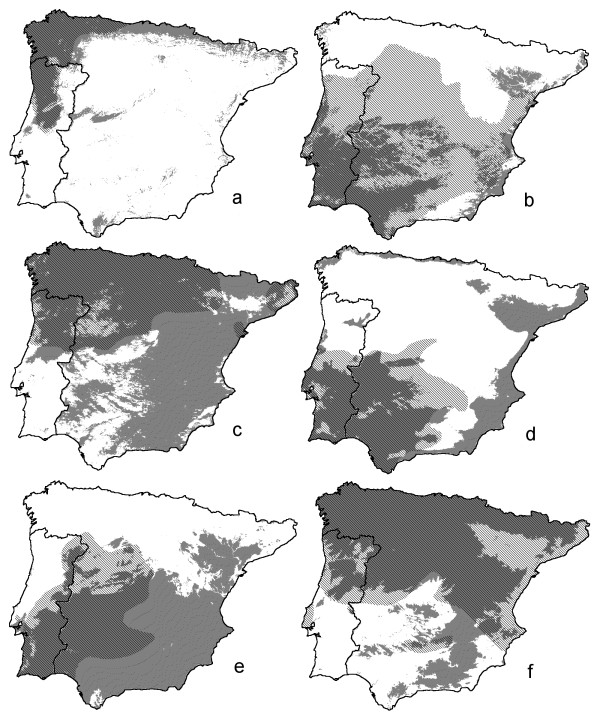
**Predictive distribution models for six amphibian species across the Iberian Peninsula**. Predictive distribution models for six amphibian species across the Iberian Peninsula, with species codes a-f as in Fig. 1. The models are based on nine environmental variables for which data are available for both Portugal and Spain (for details see Table 1). Continuous shading represents areas with a probability of occurrence g > 0.5. Hatched shading represents published range maps [37 adjusted after 38 and 39].

**Figure 4:**
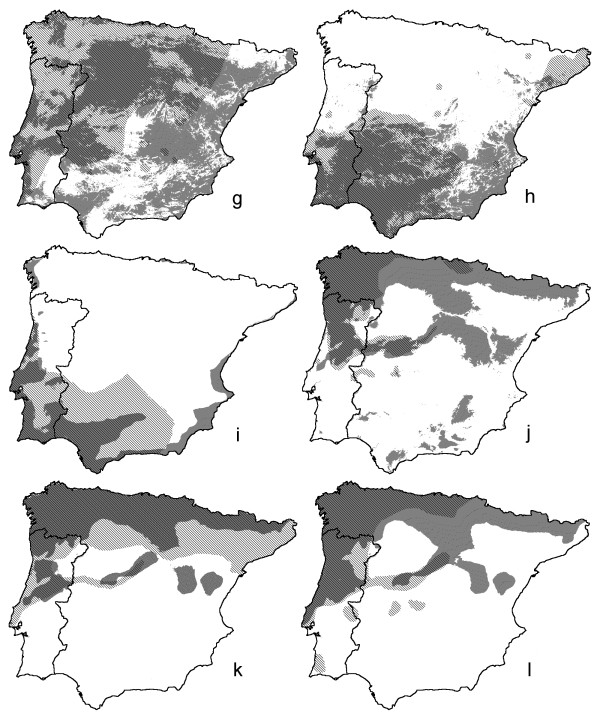
**Predictive distribution models for four amphibian and two reptile species across the Iberian Peninsula**. As Fig. 3, with four amphibian and two reptile species, with species codes g-l as in Fig. 2.

The minimum adequate models for the 12 selected amphibian and reptile edge species in Portugal (Appendix 1) included from two to nine environmental variables (Table 1, Appendix 2). Variables frequently selected in the models were, in increasing order, FROD, HARD, INSO, TJUL and PRET. Variables infrequently selected were FROM, HUMI, ACID and NDVI. Descriptive models on species distributions across Portugal are presented in Figs 1 and 2, together with documented presences. Kappa ranged from 0.35 for *H. arborea* to 0.89 for *C. lusitanica* (Table 1). Corresponding AUC values were 0.74 ± 0.021 and 0.99 ± 0.002. Following Altman [19] the strength of agreement is classified as 'fair' (0.2<k<0.4) for *P. waltl* and *H. arborea*, 'moderate' (0.4<k<0.6) for *T. pygmaeus*, *A. obstetricans*, *H. meridionalis* and *A. fragilis*, 'good' (0.6<k<0.8) for *T. marmoratus*, *A. cisternasii*, *P. ibericus*, *R. iberica* and *L. schreiberi* and 'very good' (k>0.8) for *C. lusitanica*. If nine instead of 13 environmental parameters were available for selection, kappa ranged from 0.26 to 0.86 and AUC ranged from 0.68 ± 0.023 to 0.99 ± 0.003 (Table 1). The descriptive range models for Portugal and their extrapolation over Spain are shown in Figs 3 and 4, alongside published Iberian range maps for comparison. Model fit over Spain, evaluated with range maps and expressed by kappa, was poor for *H. arborea* (kappa = 0.03), *T. marmoratus* (kappa = 0.15), *A. cisternasii* (kappa = 0.18) and *P. waltl* (kappa = 0.18), fair for *R. iberica* (kappa = 0.33), *P. ibericus* (kappa = 0.34) and *A. obstetricans* (kappa = 0.38), moderate for *T. pygmaeus* (kappa = 0.40), *L. schreiberi* (kappa = 0.49), *H. meridionalis* (kappa = 0.51) and *A. fragilis* (kappa = 0.57), and good for *C. lusitanica* (kappa = 0.61). AUC values ranged from 0.54 ± 0.010 to 0.96 ± 0.003, in approximately the same rank order as kappa (r_s _= 0.85). Model evaluation with grid cell data from UTM10 maps yielded AUC-values in the range 0.50 ± 0.010 to 0.95 ± 0.005, in approximately the same rank order as for range maps (r_s _= 0.84).

**Figure 5:**
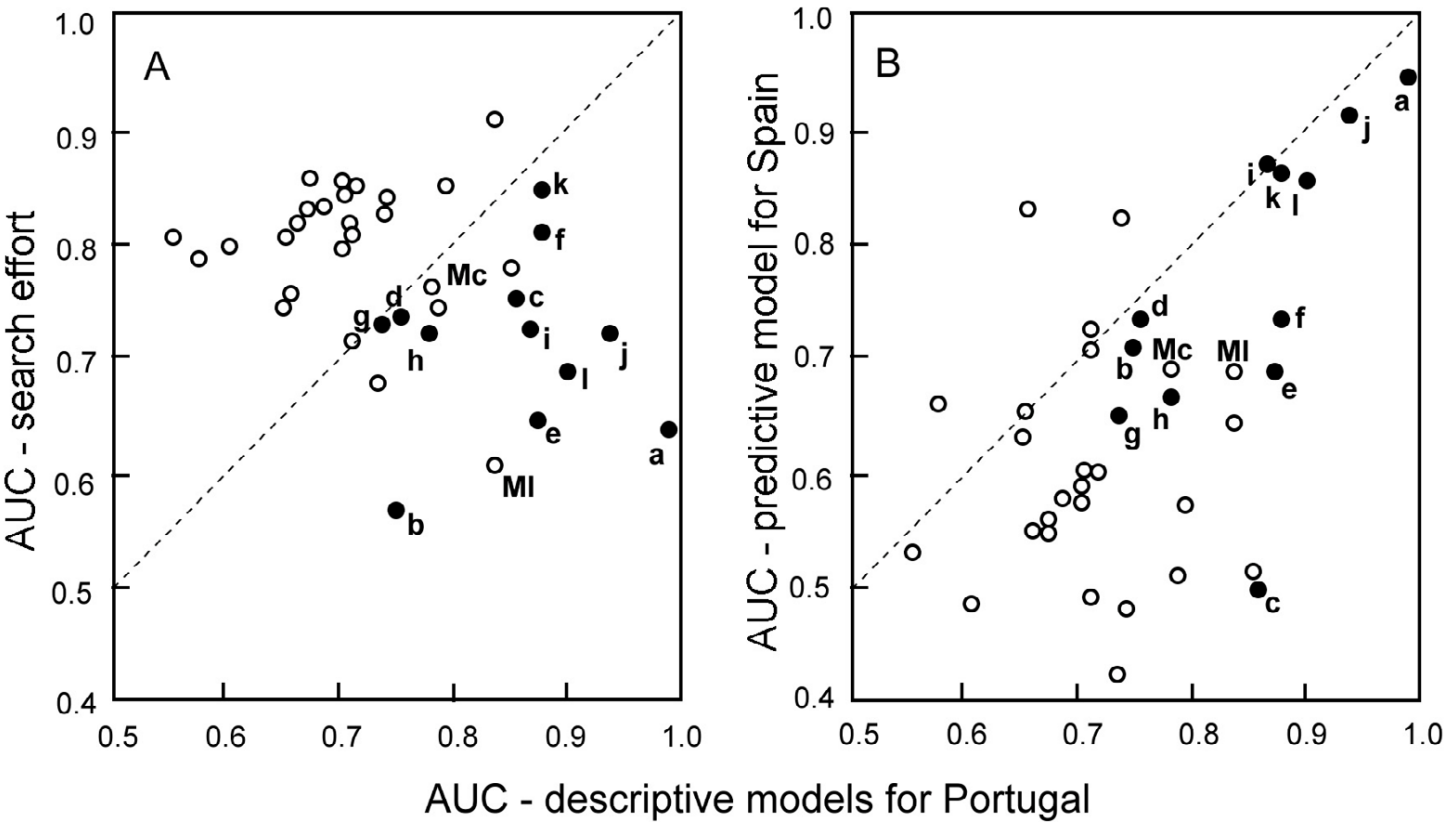
**Fit of ecographical models for Iberian amphibians and reptiles under different modelling conditions**. Bivariate plot of AUC-values, displaying the fit of distribution models for 12 edge species (solid dots, species codes as in Figs 1 and 2) and 26 countrywide species in Portugal (open dots, Mc = *Macroprotodon cucullatus*, Ml = *Mauremys leprosa*). A) Descriptive fit for models obtained with 13 environmental variables available for selection (horizontal axis) and only search-effort available for selection (E', vertical axis). Note the difference between edge- and countrywide species that have on average higher scores along the horizontal and vertical axis, respectively. B) Descriptive model fit (precision, horizontal axis, as in A) versus the fit of models extrapolated over Spain (accuracy, vertical axis). Note that precision and accuracy are significantly associated for edge species, but not for countrywide species. For statistical numerical detail see text.

Across 38 species precision averaged at kappa = 0.39 and AUC = 0.75. It was higher for the 12 edge species (group 1 – average k = 0.57, average AUC = 0.85) than for 26 countrywide species (group 2 – average k = 0.30, average AUC = 0.71). The Mann-Whithey U-test indicates that the difference is significant (kappa: Z = 4.02, P < 0.001; AUC: Z = 4.10, P < 0.001, see horizontal axis in Fig. 5A). In alternative models with search effort (E') as the one and only predictor variable available for selection model fit was similar (average k = 0.38, average AUC = 0.77), but – in contrast to the default analysis – this was due to high scores for countrywide species and low scores for edge species (Mann-Whitney U-test, Z = 3.05, P < 0.01, see vertical axis in Fig. 5A).

The correlation between model precision in Portugal and model accuracy for Spain across all species was significant (r_s _= 0.44, P < 0.01). Analysed separately, the level of significance decreased in the group of countrywide species to r_s _= -0.01 (not significant) and increased in the group of edge species to r_s _= 0.71 (P < 0.01; Fig. 5B).

## Discussion

For a given species, occupied and non-occupied areas of the globe will be separated by a – more or less abstract – generalized limit of distribution, characterized by a zone in which populations are few and far between and subject to periodic extinction [20,21]. This zone can be wide or narrow, yielding vague or sharp species borders, respectively. In vagile species such as birds, large mammals and flying insects, the range borders will tend to be wide and vague, whereas in species with low dispersal capacity, including most amphibians and reptiles, non-flying insects etc., range borders will tend to be narrow and sharp. In the current study 26 species (68%) were classified as possessing a countrywide Portuguese distribution whereas 12 species (32%) were identified as occurring in one part of the country and not the other. Other researchers, dealing with the same set of organisms, differentiate among species by 'prevalence', 'occupancy', 'extent of occurrence' (along latitudinal axis), 'marginality' and 'tolerance' and recognize four groups [see Table 2 in [17]]. My classification coincides with their 'extent of occurrence' criterion, along both (latitudinal and longitudinal) axes. More survey data, such as underway for the 'National Atlas of Amphibians and Reptiles of Portugal' (A. Loureiro et al., in prep.), support my argument for a naturally bimodal grouping at the UTM10 grid scale.

Twelve species with range borders inside the research area, i.e., Portugal, were profitably modeled from atlas data. In contrast to this, the modeled atlas data for countrywide species reflected more adequately the single variable search effort than the most informative combination out of 13 environmental variables, in 21 cases out of 26 (all open dots in the up-diagonal corner of Fig. 5A). This result indicates that the absence in a particular grid cell of a countrywide species is more likely due to inadequate sampling than that it would reflect its real absence due to local environmental conditions. The conclusion that false absences override ecological signal in these analyses may seem trivial but ecographical models for countrywide species are currently included in methodological comparisons, including approaches relying on presence and absence data [17]. I suggest that species distributed all over the area of investigation – in this study amphibians and reptiles with a countrywide Portuguese distribution – would better be excluded from such exercises, until more detailed surveying data would show firm evidence for internal species borders. This conclusion is supported by the lack of published ecographical models for countrywide species, whereas edge species are readily documented [22-24].

How to proceed from the notion that non-edge species are not adequately modeled from presence/absence data? If search effort could be controlled for, either statistically or by using a homogenous sampling effort in field studies, then presences could reflect abundance, which would be useful for modeling habitat suitability. Efforts should be made in the preparation of atlases to include information on the abundance of species instead of presenting just occurrence data, to begin with species for which observations are readily made such as widespread and locally abundant species. A proxy measure for the abundance of a species could be the effort in the terrain required to demonstrate its presence. For yet other approaches see e.g. Johnson & Sargeant [25] and Nielsen et al. [26].

Among the countrywide species, the terrapin *Mauremys leprosa* and the snake *Macroprotodon cucullatus* stand out on account of a low impact of search effort E' on the descriptive model fit and a fair predictive model performance (Fig. 5). *Mauremys leprosa* is well surveyed as part of the EC-LIFE project [27] and this work reveals a mixed distribution pattern, in which the range is continuous at UTM10 grid scale in the south and the east of the country, semi-continuous in the mid-west and, finally, with isolated occurrences in the northwest of Portugal. Its distribution appears countrywide, but from some regions it is nevertheless distinctly absent, therewith defying the edge- versus non-edge species dichotomy. The fragmented distribution pattern suggests a contracting range and obviously such a non-equilibrium situation would be difficult to model accurately. *Macroprotodon cucullatus* was not classified as an edge-species, even though it appears absent from the mid- and north-western parts of Portugal. Judgement is hampered by a low number of recordings from the regions where it does occur. Future survey data will hopefully clarify this issue.

### Environmental correlates of edge-species

The environmental variables that appear to strongly influence amphibian and reptile distributions in Portugal, for those species with a range border inside the country, are the climatic variables precipitation (PRET), insolation (INSO) and July temperature (TJUL). Parameters that appear to influence distributions less often are chemical composition of surface water (ACID), the climatic parameters describing frost conditions (FROM) and humidity and the thickness of the vegetation cover as measured by NDVI. Nevertheless, the latter parameters may have large perceived impact (values > 1 in Table 1) on particular species distributions, such as ACID in the case of *C. lusitanica*.

Species for which model fit is highest are three Iberian endemics with restricted distributions in the north of the country. However, parameter selection is not the same, indicating dissimilar ecological regimes. The generalized habitats can be summarized by high precipitation at low and medium altitudes for *C. lusitanica*, high precipitation in the case of *R. iberica* and low summer temperature and low insolation for *L. schreiberi*. These ecological descriptions at the scale of species ranges are complementary to more small-scale field observations that would characterize each of the three species as inhabiting the immediate vicinity of mountain brooks [28-30]. Although not an Iberian endemic, *A. fragilis* with a well-defined, clearly circumscribed distribution complements this series. Its generalized habitat is characterized by low summer temperature, low insolation and high precipitation. It is the only species for which the distribution model strongly suggests range subdivision over three mountainous areas separated by the rivers Douro and Mondego. Interestingly, a very similar pattern of isolation and differentiation was revealed for *C. lusitanica* with the help of molecular genetic markers [31].

The generalized habitats of five southern species can be summarized as follows: high annual temperature for *P. ibericus* and *H. meridionalis*, high annual temperature and low precipitation for *T. pygmaeus*, high annual temperature in non-mountainous terrain for *P. waltl* and low precipitation and high insolation in the case of *A. cisternasii*. The small-scale, local habitats of the five species are not broadly overlapping, either through a differential focus on ephemeral ponds (*P. ibericus*), permanent ponds (*T. pygmaeus*) and streams (*A. cisternasii*) for breeding and pre-metamorphic life, or by a differential habitat preference at the post-metamorphic stage, that is either largely terrestrial (*H. meridionalis*) or largely aquatic (*P. waltl*).

Three species have large northern distributions reaching central France (*T. marmoratus*), the south of the Netherlands (*A. obstetricans*) and southern Sweden (*H. arborea*) combined with the presence of a congeneric species at the southern edge of the range (*T. pygmaeus*, *A. cisternasii* and *H. meridionalis*, respectively). The *T. marmoratus* distribution in Portugal appears to be impacted by a low degree in hardness of the water. The *A. obstetricans* distribution is characterized by high altitude, high precipitation, low insolation and low water hardness. Finally, the distribution model for *H. arborea* combines the highest number of selected variables (nine) with the lowest distribution model fit (kappa 0.35). This suggests either that one or more ecological parameters crucial to the species are not incorporated in the analysis (e.g. particular resources, the presence of a congeneric competitor) or that the ecological requirements of the species are diverse, difficult to model, perhaps even shift across the range [32]. Another possibility is that *Hyla arborea* is, like *Mauremys leprosus*, a species not amenable to contemporary modeling due to a contracting range.

**Table 1:** Distribution models for ten amphibians and two reptiles by logistic regression analysis of presence-absence data for Portugal. Model conditions are with 13 variables (condition 1) or with nine variables (condition 2; details see text). Environmental data are standardized, except for LITH. Paramers with large effect (values >1) are shown in boldface type. The fit of the descriptive models is expressed by Cohen's kappa and the 'Area Under the Curve in Receiver Operating Characteristic' plots (AUC) and asymptotic standard error (SE). Distribution models derived from the equations are shown as 'probability of occurrence' maps for Portugal in figures 1 and 2 and as range maps for the Iberian Peninsula in figures 3 and 4.

	Observed	Modelling	Model equation †															Model fit	
				
Species	presences	condition	ACID	ALTI	FROD	FROM	HARD	HUMI	INSO	LITH ‡		NDVI	PRET	RELI	TEMP	TJUL	Constant	kappa	AUC ± SE
			
Amphibians																			
a) *Chioglossa lusitanica*	202	1	**-1.120**	**-1.635**									**3.405**	**1.919**		**-1.226**	-2.511	0.8847	0.989 ± 0.002
		2		**-1.864**									**3.900**	**1.743**	**-1.170**		-1.857	0.8547	0.987 ± 0.003
b) *Pleurodeles waltl*	144	1, 2												-0.803	0.848		-0.559	0.3885	0.745 ± 0.018
c) *Triturus marmoratus*	226	1					**-1.406**			-1.057	-0.316			0.671			-0.368	0.6305	0.853 ± 0.013
		2						-0.522	-0.611			-0.780	0.607		-0.565		-0.649	0.596	0.854 ± 0.013
d) *T. pygmaeus*	130	1, 2			-0.512				-0.652			0.253	-0.943		0.984	0.444	-0.796	0.4072	0.751 ± 0.018
e) *Alytes cisternasii*	223	1			-0.623			-0.411	**1.066**	-1.376	0.759		**-1.210**				-0.915	0.5967	0.871 ± 0.012
f) *A. obstetricans*	169	1		0.872			-0.617		-0.416				0.535				-0.836	0.5737	0.879 ± 0.013
		2		**1.028**					-0.554				0.610				-0.674	0.5581	0.879 ± 0.013
g) *Hyla arborea*	132	1	-0.458		-0.523	0.336	-0.404	-0.367		0.789	-0.538	0.486	-0.849			-0.631	-0.337	0.3533	0.742 ± 0.021
		2			-0.463			-0.335					-0.526	-0.480	-0.615	-0.386	-0.167	0.2575	0.682 ± 0.023
h) *H. meridionalis*	95	1, 2		0.972	-0.577				0.408					-0.707	**1.115**		-0.709	0.4301	0.777 ± 0.02
i) *Pelodytes ibericus*	91	1			-0.371	-0.604	0.785								**1.753**	-0.717	-1.451	0.5909	0.863 ± 0.016
		2			-0.738										**2.533**	-0.639	-1.446	0.5052	0.843 ± 0.018
j) *Rana iberica*	229	1		0.635			-0.902						**1.988**				-0.934	0.7448	0.936 ± 0.008
		2		0.753					-0.605				**1.890**				-0.746	0.7494	0.935 ± 0.008
Reptiles																			
k) *Anguis fragilis*	83	1, 2						-0.360	-0.605				0.512			**-1.046**	-1.141	0.5866	0.876 ± 0.016
l) *Lacerta schreiberi*	272	1				-0.273			**-1.080**	-1.239	-0.501					**-1.327**	-0.103	0.6441	0.899 ± 0.010
		2							-0.858				0.479			**-1.104**	-0.594	0.6353	0.897 ± 0.010

†) Model equation for e.g. *Chioglossa lusitanica *under model conditions of type 1 is: probability of occurrence = (1/(1+exp(1.120*ACID+1.35*ALTI-3.405*PRET-1.919*RELI+1.226*TJUL+2.511))).

‡) The categorical variable 'LITH' Is represented by two binary variables.

## Conclusion

Among edge-species, a statistically significant relationship was found between precision and accuracy of the models, that is, between model fit over documented and extrapolated parts of the range. At one side of the continuum, distribution models with low precision are unlikely to help much in the identification of the ecological parameters underlying species distributions. The information extracted from poor modelling results is unlikely to be useful and should not be made operational. Improved models may be obtained through a biologically better informed selection of explanatory variables [cf. [33]]. On the other side of the continuum of low versus high model performance, well-fitting descriptive models do tend to predict distributions rather well. Such models yield biologically meaningful information, potentially leading to an improved understanding of species' ecological requirements – information that subsequently can be made to use in conservation management. At the scale of the amphibians and reptiles of Portugal this condition however applies to a minority of the species.

## Methods

### Biological data

Distribution data on the Portuguese herpetofauna (18 amphibian and 27 reptile species) organised in Universal Transverse Mercator (UTM) grid cells with a spatial resolution of 10 × 10 km were taken from Godinho et al. [34]. This is basically the work of Malkmus [28] upgraded with data from the literature, theses, technical reports, single species accounts, etc. Species were *a priori* divided in two groups of (1) edge species, that show a range border inside continental Portugal and (2) countrywide (or non-edge) species, which do not show a range border inside continental Portugal. This distinction was made to test the hypothesis that the spatial-environmental modelling of atlas data is only potentially meaningful if at least some of the reported absences are definitely real (as in group 1 species). Conversely, presented absences for group 2 species could – ultimately – all be 'false absences' (grid cells for which the species was not recorded despite its presence).

Twelve edge species in group 1 are the salamanders *Chioglossa lusitanica* and *Pleurodeles waltl*, the frogs *Hyla arborea*, *H. meridionalis*, *Pelodytes ibericus* and *Rana iberica*, the toads *Alytes cisternasii* and *A. obstetricans* and the lizards *Anguis fragilis* and *Lacerta schreiberi*; sufficient data were also available for two fairly cryptic newt species *Triturus marmoratus* and *T. pygmaeus* following the reconstruction of the contact zone between them in central Portugal (JWA & G. Themudo, unpublished data). The lizard '*Podarcis bocagei*' incorporates a cryptic species and both were excluded due to low record numbers [35]. Twenty-six non-edge species in group 2 are two salamanders, two frogs, three toads, two terrapins, nine lizards and eight snakes (for species names see Appendix 1). Grid cells for which less than 50% of the coverage was for continental Portugal and species with less than 50 grid cell records (n = 7, Appendix 1) were excluded from the analyses [36]. The amphibian and reptile distribution data for Spain used to evaluate the models were species range maps [37-39] and atlas data organized in the UTM10-grid [40].

Search effort is defined as E=(N_obs_/N_max_)^2^, with N_obs _= the number of species actually observed in a grid cell and N_max _= the maximum number of species that could be observed in that grid cell, according to atlas data and range maps, respectively. Search effort was calculated for each relevant grid cell of the Iberian Peninsula. E' was used as a measure of search effort uncoupled from the species under consideration, that is, with data for that species excluded. These functions incorporate the notion that cells with greater surveying effort will have more species reported, but that, once the effort becomes substantial, new species will be added only slowly.

### Environmental data

Twenty-one ecologically meaningful environmental parameters were pre-selected for analysis. For 18 variables information was available in digital format for Portugal [41]. A vegetation map (normalised difference vegetation index or NDVI) was obtained courtesy of the Royal Dutch Meteorological Institute (KNMI). An altitude map was taken from the internet  and used to produce a relief map by a set of filter operations [42]. Maps of the mean January and July temperatures were digitised from the Portuguese climate atlas [43]. Variables were (de)selected using criteria of i) redundancy at r_s _>0.8, ii) availability for both Portugal and Spain and iii) promise in terms of amphibian and reptile life history. The following 13 variables were retained in three groups : climatic variables – annual precipitation (PRET, in mm), humidity of the air (HUMI, in %), average temperature over the year (TEMP, in °C) and for July (TJUL, in °C), annual number of frost days (FROD, in days), annual number of frost months (FROM, in months) and insolation (INSO, in hours); *topographical variables* – altitude (ALTI, in m asl), relief (RELI, in arbitrary scale) and *geological/geochemical* variables – acidity of surface water (ACID, pH in 14 classes), hardness of the water (HARD, CaCO3 mg/l in 17 classes), lithology (LITH, in the classes, sedimentary, sedimentary and metamorphic and igneous). Finally, the vegetation index (NDVI) may be seen as belonging to the latter group of variables. For detail on these candidate predictor variables see Appendix 2. Data for Spain were digitised from the Atlas Nacional de España [44]. All variables (except LITH) were standardized to an average of zero and standard deviation of unity, to increase the comparability of their effects. The variables were introduced into the GIS analytical software as raster layers with 1 km spatial resolution. Mean values for 10 × 10 km UTM grid cells were obtained by averaging the data (modal values for the categorical variable LITH).

### Analysis and modelling

Logistic regression analyses were performed with SPSS 12 [45] with a forward stepwise addition of independent variables and with Bonferroni correction to the initial α = 0.05 [46]. The impact of presences and absences was equilibrated through a weighting variable, using the 'weight cases' option in SPSS. Thus, the outnumbering case, either presences or absences, was down weighted to obtain a balanced dataset with effectively a fifty/fifty distribution of presence and absences. Extrapolation of the models to Spain was performed on the basis of nine environmental variables for which data were available (all but ACID, FROM, HARD and LITH). The strength of agreement among distribution data and distribution models was summarized with Cohen's kappa [47], using a 50% threshold, and with 'Area Under the Curve' statistics (AUC), determined from so-called Receiver Operated Character plots [48,49] in SPSS.

## Supplementary Material

Appendix 1 Click here for file

Appendix 2 Click here for file
