# Supplementary material for: From descriptive to predictive distribution models: a working example with Iberian amphibians and reptiles
Source: Front Zool. 2006 May 4;3:8. doi: 10.1186/1742-9994-3-8 (PMC1534039; doi:10.1186/1742-9994-3-8)
Supplement: Appendix 1 [file 1742-9994-3-8-S1.doc]

Appendix 1 - Amphibian and reptile species observed in continental Portugal, after Godinho et al. [34].

Group 1, with species border inside the country

Salamanders

Chioglossa lusitanica Bocage, 1864

Pleurodeles waltl Michahelles, 1830

Triturus marmoratus (Latreille, 1800)

T. pygmaeus (Wolterstorff, 1905)

Frogs and toads

Alytes cisternasii Boscá, 1879

A. obstetricans (Laurenti, 1768)

Hyla arborea (Linnaeus, 1758)

H. meridionalis Boettger, 1874

Pelodytes ibericus Sánchez-Herráiz, Barbadillo, Machordom & Sanchíz, 2000

Rana iberica Boulenger, 1879

Lizards

Anguis fragilis Linnaeus, 1758

Lacerta schreiberi Bedriaga, 1878

Group 2, with countrywide distributions

Salamanders

Salamandra salamandra (Linnaeus, 1758)

Triturus boscai (Lataste, 1879)

Frogs and toads

Bufo bufo (Linnaeus, 1758)

B. calamita (Laurenti, 1768)

Discoglossus galganoi Capula, Nascetti, Lanza, Bullini & Crespo, 1985

Pelobates cultripes (Cuvier, 1829)

Rana perezi Seoane, 1885

Terrapins

Emys orbicularis (Linnaeus, 1758)

Mauremys leprosa (Schweigger, 1812)

Lizards

Acanthodactylus erythrurus (Schinz, 1833)

Blanus cinereus (Vandelli, 1797)

Chalcides bedriagai (Boscá, 1880)

Chalcides striatus (Cuvier, 1829)

Podarcis hispanica (Steindachner, 1870)

Psammodromus algirus (Linnaeus, 1758)

P. hispanicus Fitzinger, 1826

Tarentola mauretanica (Linnaeus, 1758)

Timon lepidus (Daudin, 1802)

Snakes

Coluber hippocrepis Linnaeus, 1758

Coronella girondica (Daudin, 1803)

Elaphe scalaris (Schinz, 1822)

Macroprotodon cucullatus (Geoffroy Saint-Hilaire, 1827)

Malpolon monspessulanus (Hermann, 1804)

Natrix maura (Linnaeus, 1758)

Natrix natrix (Linnaeus, 1758)

Vipera latasti Boscá, 1878

Group 3, deselected due to low record number (N<50)

Salamanders

Triturus helveticus (Razoumowsky, 1789)

Lizards

Archaeolacerta monticola (Boulenger, 1905)

Chamaeleo chamaeleon (Linnaeus, 1758)

Hemidactylus turcicus (Linnaeus, 1758)

Podarcis bocagei (Seoane, 1884)

Snakes

Coronella austriaca Laurenti, 1768

Vipera seoanei Lataste, 1879
