# Supplementary material for: From descriptive to predictive distribution models: a working example with Iberian amphibians and reptiles
Source: Front Zool. 2006 May 4;3:8. doi: 10.1186/1742-9994-3-8 (PMC1534039; doi:10.1186/1742-9994-3-8)
Supplement: Appendix 2 [file 1742-9994-3-8-S2.doc]

| Appendix 2 – Environmental parameters used to model the distribution of golden-striped salamander in Portugal, after Teixeira & Arntzen [24] | | | |
| --- | --- | --- | --- |
|  |  |  |  |
| Variable | Code | Selected for analysis | Available for Spain |
|  |  |  |  |
| Acidity of the soil (pH) | ACID | yes | no |
| Altitude (m) | ALTI | yes | yes |
| Chlorates content of subterranean water (Cl^-^ mg/l) | CHLO | no | no |
| Water drainage (mm/year) | DRAI | no | no |
| Evapotranspiration (mm/year) | EVAP | no | yes |
| Frost days (number/year) | FROD | yes | yes |
| Frost months (number/year) | FROM | yes | no |
| Hardness of subterranean water (CaCO_3_ mg/l) | HARD | yes | no |
| Humidity of the air (%) | HUMI | yes | yes |
| Insolation (hours) | INSO | yes | yes |
| Lithology (sedimentary, metamorphic, igneous) | LITH | yes | no |
| Vegetation index | NDVI | yes | yes |
| Precipitation days (days/year) | PRED | no | yes |
| Annual total precipitation (mm/year) | PRET | yes | yes |
| Solar radiation (Kcal/ cm^2^) | RADI | no | yes |
| Slope (%), produced from ALTI | RELI | yes | yes |
| Residual content of subterranean water (mg/l) | RESI | no | no |
| Sulphates content of subterranean water (SO_4_^-2^ mg/l) | SULP | no | no |
| Annual mean temperature (ºC) | TEMP | yes | yes |
| January mean temperature (ºC) | TJAN | no | yes |
| July mean temperature (ºC) | TJUL | yes | yes |
